# The Cost-Effectiveness of Directly Observed Highly-Active Antiretroviral Therapy in the Third Trimester in HIV-Infected Pregnant Women

**DOI:** 10.1371/journal.pone.0010154

**Published:** 2010-04-13

**Authors:** Caitlin J. McCabe, Sue J. Goldie, David N. Fisman

**Affiliations:** 1 Child Health Evaluative Sciences, Research Institute of the Hospital for Sick Children, Toronto, Ontario, Canada; 2 Department of Health Policy and Management, Harvard School of Public Health, Boston, Massachusetts, United States of America; 3 Department of Health Policy, Management and Evaluation, University of Toronto, Toronto, Ontario, Canada; 4 Dalla Lana School of Public Health, University of Toronto, Toronto, Ontario, Canada; University of Toronto, Canada

## Abstract

**Background:**

In HIV-infected pregnant women, viral suppression prevents mother-to-child HIV transmission. Directly observed highly-active antiretroviral therapy (HAART) enhances virological suppression, and could prevent transmission. Our objective was to project the effectiveness and cost-effectiveness of directly observed administration of antiretroviral drugs in pregnancy.

**Methods and Findings:**

A mathematical model was created to simulate cohorts of one million asymptomatic HIV-infected pregnant women on HAART, with women randomly assigned self-administered or directly observed antiretroviral therapy (DOT), or no HAART, in a series of Monte Carlo simulations. Our primary outcome was the quality-adjusted life expectancy in years (QALY) of infants born to HIV-infected women, with the rates of Caesarean section and HIV-transmission after DOT use as intermediate outcomes. Both self-administered HAART and DOT were associated with decreased costs and increased life-expectancy relative to no HAART. The use of DOT was associated with a relative risk of HIV transmission of 0.39 relative to conventional HAART; was highly cost-effective in the cohort as a whole (cost-utility ratio $14,233 per QALY); and was cost-saving in women whose viral loads on self-administered HAART would have exceeded 1000 copies/ml. Results were stable in wide-ranging sensitivity analyses, with directly observed therapy cost-saving or highly cost-effective in almost all cases.

**Conclusions:**

Based on the best available data, programs that optimize adherence to HAART through direct observation in pregnancy have the potential to diminish mother-to-child HIV transmission in a highly cost-effective manner. Targeted use of DOT in pregnant women with high viral loads, who could otherwise receive self-administered HAART would be a cost-saving intervention. These projections should be tested with randomized clinical trials.

## Introduction

Mother-to-child transmission is the most common cause of pediatric human immunodeficiency virus (HIV) infection in North America. United States surveillance data identified 139 newly recognized HIV infections in children of mothers with HIV infection in the U.S. in 2007; over 9,500 children with likely vertical acquisition of HIV infection have developed AIDS since the HIV epidemic began [1]. Maternal viral load in the third trimester of pregnancy is a strong predictor of risk of HIV transmission to the newborn, and its reduction through the use of highly-active antiretroviral therapy (HAART) appears to markedly reduce the risk of peripartum HIV transmission [2], [3], [4], [5].

Recognition of the large potential impact of maternal HIV replication on transmission has resulted in revision of U.S. and European guidelines on antiretroviral use in pregnancy [6], [7], [8], [9]. These guidelines have long advocated the use of HAART in pregnancy for prevention of transmission, and HAART is now the standard of care for all pregnant HIV-infected women [6]. Adherence to a HAART regimen predicts suppression of viral replication and reduces the potential for emergence of resistant viral strains [10], [11], [12], [13]. Drug adherence is suboptimal in up to one-third of HIV-infected women of childbearing age [14], [15], [16], [17], notwithstanding increases in HAART compliance during pregnancy; mother-to-child transmission still occurs in 1–3% of infants born to HIV-infected women receiving antiretroviral treatment [17]. As low adherence to anti-retroviral therapies is significantly associated with mortality and increased risk of mother-to-child transmission [16], [17], it is important to establish other means of improving adherence in this high-risk group in order to improve clinical outcomes in both mothers and their infants.

Directly observed therapy (DOT) is used in the treatment of tuberculosis to enhance the likelihood of treatment success and minimize the emergence of drug-resistant microbes, and has been proposed as a means of improving adherence to HAART [18], [19]. With HIV, unlike tuberculosis, disease duration is life-long, and medications must be given daily, making the routine use of directly observed HAART problematic. Recent randomized trials have demonstrated significant reductions in viral load in individuals receiving DOT for HIV infection, although this response may not be durable after discontinuation of DOT [20], [21], [22]. Nonetheless, in prisons and community-based settings, the use of DOT for HIV infection has been associated with a reduction in viral burden of 1 to 2 log, even in antiretroviral-experienced individuals [19], [20], [23], [24], [25], [26].

The third trimester of pregnancy may present a unique opportunity for use of directly observed HAART in that the time horizon is limited. In addition to virologic suppression for the prevention of mother-to-child transmission of HIV, directly observed HAART could preserve the future potency of antiretroviral drug classes by limiting emergence of resistance in pregnant women taking these medications solely to prevent transmission. Furthermore, DOT could potentially be an economically attractive intervention, since, if successful, downstream medical costs associated with pediatric HIV infection will be prevented.

The small number of perinatally infected infants makes it challenging to study the impact of DOT-HAART for prevention of perinatal HIV transmission in the context of a prospective, randomized trial; in the absence of such a trial, simulations that aggregate the best available data may be a useful tool to optimize policy under uncertainty. Our objective was to use a simulation model to project the costs and clinical benefits that would be expected to result from the use of DOT in women receiving HAART in the third trimester of pregnancy.

## Methods

### The Model

**Figure 1:**
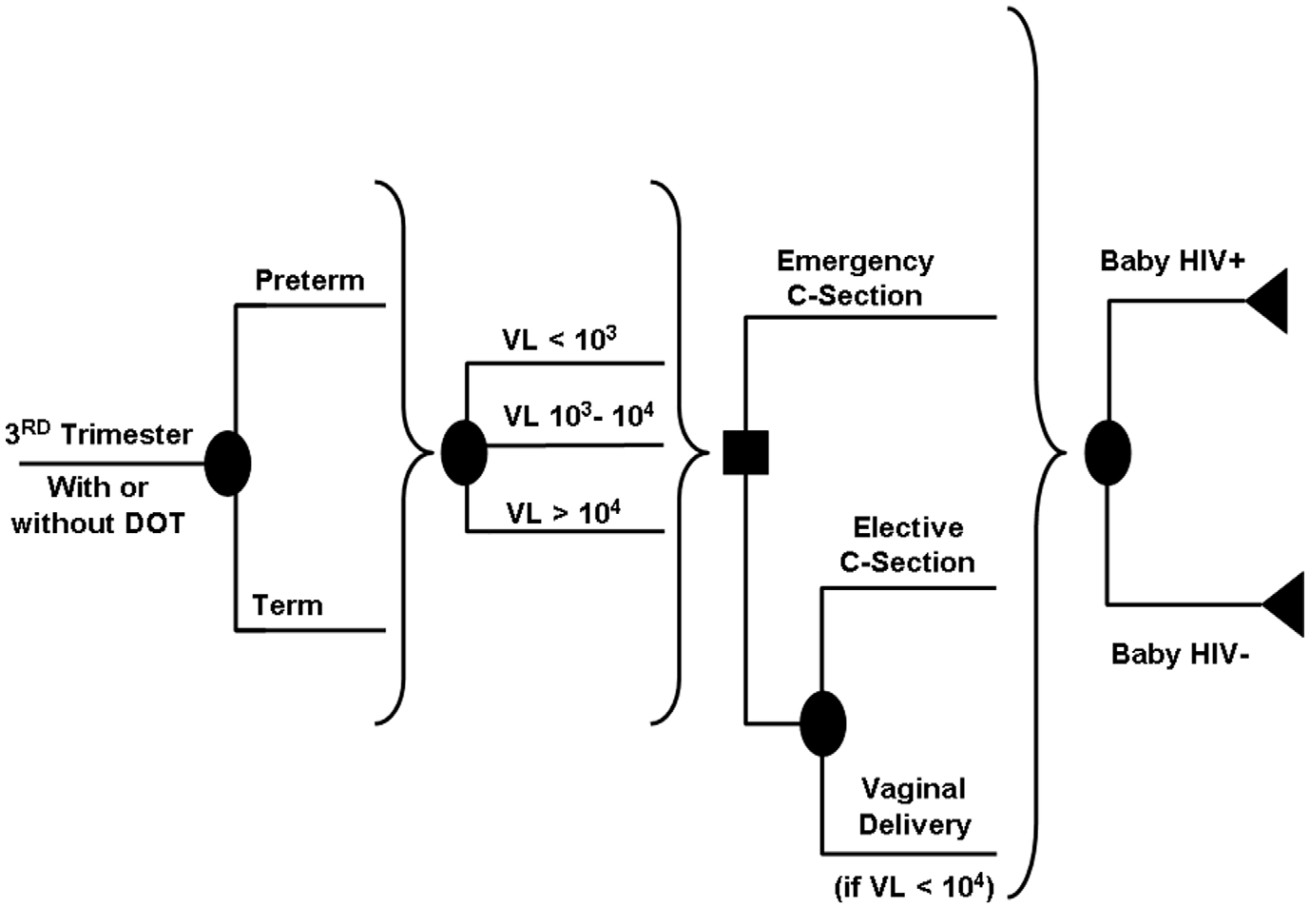
Simplified Depiction of Model Tree Structure. Pregnant women with HIV infection enter the third trimester of pregnancy already on highly-active antiretroviral therapy, with or without direct observation. Round “nodes” represent chance events, while squares represent clinical decisions. Women either experience preterm or term delivery, with a viral load (VL, in copies/ml) that is a function of baseline viral load, effectiveness of antiretroviral drugs, and the availability of directly observed therapy. For both detectable and undetectable viral load responses, a proportion of women receive emergency Caesarean sections. Delivery may otherwise be by elective Caesarean section, or by vaginal delivery. Vaginal delivery is an option only in women with low viral loads on antiretroviral therapy. Health outcomes in the infant are predicted by prematurity (not shown) and the occurrence of mother-to-child transmission.

We constructed a probabilistic model of HIV infection in pregnancy to project the impact of directly observed HAART on vertical HIV transmission by HIV-infected women receiving treatment by the 3^rd^ trimester of pregnancy (before 28 weeks gestation) according to current U.S. Department of Health and Human Services guidelines [6] (**Figure 1**), with some proportion of these women having started HAART prior to pregnancy for their own health, and some proportion starting within the first two trimesters of pregnancy with the goal of preventing mother-to-child HIV transmission. Clinical and cost outcomes were projected for a hypothetical cohort of women with asymptomatic HIV infection and CD4-positive lymphocyte counts >200 copies/ml.

Women receiving HAART enter the model in the 3^rd^ trimester of pregnancy. We assumed that all transmission occurs in the third trimester of pregnancy or in the peripartum period, and that the probability of transmission is conditional on the maternal viral load at the time of parturition. We incorporated the risk of preterm birth and the subsequent elevated risk of neonatal death, and the relative risks associated with emergency and elective Caesarean section and vaginal delivery. We assumed all infants would be formula-fed, making acquisition of HIV through breast feeding unlikely [27], [28].

### Strategies

We used our model to project downstream costs and consequences associated with self-administered HAART and directly observed HAART. For both strategies we assumed patients would receive otherwise standard obstetrical management. HAART was assumed to consist of a combination of two nucleoside reverse transcriptase inhibitors (excluding the combination of didanosine and stavudine) and one or two viral protease inhibitors, with the regimen dosed one or two times daily [6]. We assumed that non-nucleoside reverse transcription inhibitors would not be routinely used for longitudinal therapy in the third trimester [6]. Although current guidelines recommend that all women receive combination antiretroviral therapy in pregnancy, for their own health and/or to prevent vertical transmission [6], we added a hypothetical “no HAART” arm by removing the virological benefits and potential toxicity of HAART, in order to evaluate the cost-effectiveness of both antiretroviral strategies relative to no treatment.

DOT was assumed to consist of daily home visits by a trained public health worker, with observation of the first dose of antiretroviral drugs. If a second daily dose was required, it would be self-administered, as described elsewhere [19]. Standard obstetrical management was considered to consist of monthly visits in the third trimester. Elective Caesarean section delivery was assumed to occur before the rupture of membranes and onset of labour, and was associated with a reduced risk of HIV transmission. Emergency Caesarean delivery was operative delivery after the rupture of membranes and/or onset of labor, and was not associated with a reduced risk of transmission. We assumed that elective Caesarean section was performed in women with viral loads >1,000 copies/ml at term not requiring emergency Caesarean section [6]. Where viral load was <1,000 copies/ml at term and emergency Caesarean section was not required, women could receive either elective Caesarean section or a vaginal delivery. We explored the costs and benefits associated with targeted use of DOT among certain subgroups of women.

### Parameterization and Calibration

Probabilities and ranges used in the model were derived from published medical literature obtained through keyword searches on MEDLINE, and through consultation with expert clinicians involved in treatment of pregnant women with HIV infection (**Table 1**). Individual women's baseline viral load “set points” (i.e., the approximate viral load that would be expected in that individual in the absence of antiretroviral therapy [29]) were drawn at random from a log-normal distribution. The baseline viral load distribution was constructed using viral loads observed in a cohort of 180 antiretroviral-naïve men [30], and reduced by 0.25 log to account for the possibility that viral set point is lower in women [31]. The impact of self-administered HAART was simulated by reducing the baseline viral load using data on the effectiveness of HAART from the Women's Interagency HIV Study (WIHS) [32]. In women assigned to DOT, viral load was reduced again by a log quantity drawn randomly from a triangular distribution, to simulate the increase in adherence reported in association with directly observed HAART [19], [23], [24], [25]. In the base case, we assumed that toxicity secondary to HAART in the developing infant would not be enhanced by directly observed HAART.

**Table 1:** Selected Model Variables.

Variable	Value (Range)	Reference
Baseline log_10_ viral load (±SD)	3.75 (±0.8)	[30], [31]
Probability of virologic response to HAART without DOT *		
Complete response	0.35 (0–1)	[32] and calibration
Partial response	0.42 (0–0.65)	[32] and calibration
Non-response	0.23 (0–0.5)	[32] and calibration
Log_10_ reduction in viral load with DOT	0.8 (0.1–1.5)	[20], [21], [22]
Probability of non-elective Caesarean section †	0.16	[59] and calibration
Probability of Caesarean section (elective and non-elective)	0.60	[60] and calibration
Probability of premature delivery † ‡	0.2 (0.06–0.27)	[58], [61]
Probability of antiretroviral toxicity §	0 (0.005–0.05)	[6], [62], [63], [64], [65]
Probability of vertical transmission of HIV ∥		
Maternal viral load >10,000 copies/ml	0.21 (0.13–0.28)	[2], [5] and calibration
Maternal viral load 1,000–10,000 copies/ml	0.13 (0.06–0.21)	[2], [5] and calibration
Maternal viral load <1,000 copies/ml	0.0015 (0–0.002)	[2], [5] and calibration
Relative risk of transmission with elective Caesarean section	0.4 (0.2–0.6)	[59], [66]
Relative risk of drug toxicity with DOT	1 (1–5)	[67] and best estimate
Discounted QALY, infant ¶		
HIV uninfected	28 (28–29)	
HIV infected **	9 (8–15)	
Reduction in QALE (%)		
Prematurity ††	20 (15–30)	[68], [69], [70]
Drug toxicity	0 (10–40)	Best estimate
Costs		
HAART x one trimester	$4890 ($3750–$5640)	[6], [71], [72]
Peripartum zidovudine ‡‡	$550 ($410–$740)	[6], [72]
Directly observed therapy	$2630 ($2630–$21,000)	[45], [46]
Vaginal delivery	$3610 ($2370–$10,380)	[38], [73]
Caesarean section	$6570 ($5260–$15,110)	[38], [73]
Lifetime costs, prematurity ¶ §§	$278,600 ($214,200–$407,400)	[68], [74], [75], [76]
Lifetime healthcare costs, drug toxicity	$0 ($65,700–$262,800)	Best estimate
Lifetime healthcare costs, pediatric HIV infection ¶	$289,000 ($140,600–$660,930)	[38], [40], [41], [42], [43], [44], [77]
Discount rate (%)	3 (0–10)	[48]

**NOTE:** HAART, highly-active antiretroviral therapy; DOT, directly observed therapy; QALE, quality-adjusted life expectancy.

* Complete response considered to be a sustained reduction in viral load of 2.0 log_10_ copies/ml; partial response considered to be a sustained reduction of 0.75 log_10_ copies/ml; non-response associated with a reduction of 0.25 log_10_ copies/ml [32].

† Non-elective Caesarean at term and Caesarean section with premature delivery were not associated with reduced risk of mother-to-child HIV transmission [59].

‡ Base-case probability of prematurity approximates that seen in a cohort of HIV-infected New York Medicaid recipients; upper bound based on rates of premature delivery seen in a subgroup of women receiving methadone maintenance therapy [58].

§ In base case, risk of antiretroviral toxicity in infants was assumed to be negligible, consistent with available data [6], [62], [65]. Risk of severe toxicity used in sensitivity analysis based on upper bound confidence limit for mitochondrial toxicity in a French cohort study [63], [64]. Risk of moderate toxicity based on best estimate of plausible upper bound.

∥ Base-case estimates and ranges for viral loads greater than 1000 copies/ml derived based on outcomes among individuals receiving peripartum zidovudine in a prospective multi-centre study of HIV in pregnancy [5].

¶ QALY and lifetime cost estimates presented in table based on the use of a 3% discount rate. Base-case quality-adjustment for HIV-uninfected individuals 45 years of age and older performed using community-derived utility estimates, as described in [78], while upper bound estimates are not quality-adjusted.

** Quality-adjusted survival in HIV-infected infants was estimated based on the assumption that 2/3 of person-time with HIV infection would be symptom-free, while 1/3 of person-time with HIV would include HIV-attributable symptoms. Acquired immune deficiency syndrome was assumed to be present in the last two years of life. Death due to HIV in infected children has declined markedly in both the U.S. and Europe with the advent of HAART, making estimation of survival in HIV-infected children difficult due to small numbers of events [35], [37]. Survival in HIV-infected children was assumed to approximate that seen in the youngest adults treated with HAART [36]. Lower bound survival estimates for HIV were generated using community-derived utility weights for life with HIV infection [34], while upper bound estimates were generated using more favorable survival estimates, and without quality-adjustment [79].

†† Based on in-hospital mortality in 15% of premature infants (including third-trimester still-births), with a risk of moderate to severe cognitive impairment in 10–30% [68], [70], [76]. Reduction in quality-adjusted survival estimated based on health utility weight of 0.67 predicted for an individual with moderate cognitive and sensory impairment and impaired self-care ability using the Health Utilities Index Mark II [69].

‡‡ Based on intravenous zidovudine during 12 hour labor, and average dose of 1 ml zidovudine syrup (10 mg/ml) administered to neonate qid for 6 weeks postpartum [6].

§§ Estimated based on weighted average healthcare costs associated with prematurity in infants born from 28 to 36 weeks of gestation in the state of California [75], with future costs occurring due to developmental delay in 15–30% of surviving infants [68], [74], [76], [80].

To ensure model validity, we calibrated parameters to so that outcomes would approximate those observed in a 2005 European Collaborative Study that showed the effectiveness of HAART in preventing mother-to-child HIV transmission in a cohort study of over 4000 mother-child pairs [33]. We calibrated values for the proportion of women with a viral load <200 copies/ml and the probability of vertical transmission at all viral loads, and we empirically inflated the rates of Caesarean sections (both emergency and elective) and premature births to more closely match the European study's proportions.

Our primary outcome of interest was the quality-adjusted life expectancy (QALY) of infants born to women with HIV infection. Quality-adjusted survival estimates in HIV-infected children incorporated patient-derived, preference-based utility measures [34], in combination with survival estimates reported for pediatric populations and (because most infants will survive to adulthood) adult populations since the advent of HAART [34], [35], [36], [37]. Intermediate outcomes of interest included the number of Caesarean sections performed, and the number of HIV infections in infants under each strategy.

We estimated costs from a societal perspective, including future healthcare costs associated with new HIV infections in infants [38], [39], [40], [41], [42], [43], [44]. The cost of delivery and direct observation of antiretroviral therapy was assumed to be equivalent to that described for tuberculosis-related directly observed therapy [45], [46]. All costs were converted to 2008 U.S. dollars using the Consumer Price Index for Medical Care Services [47], and future costs were discounted using a 3% annual rate in the base case [48].

A health care intervention is considered to be “cost-saving” when it costs less but provides incremental benefit relative to a competing intervention; “highly cost-effective” when it costs less than the GDP per capita; and “cost-effective” when it is between one and three times a country's GDP per capita [49]. A cost-saving intervention is always preferred to competing programs or strategies [49], [50].

### Simulations

We generated estimates of costs and consequences of competing strategies through a series of Monte Carlo simulations. Such simulations utilize a random-number generator to create unique, simulated individual patients, and move them through a series of chance events over time [51], [52]. A running tally of outcomes, costs, and events is recorded, with the creation of simulated cohorts that can be compared to one another. Unless otherwise stated, results presented here are each based on five thousand simulations of 200-person clinical trials, with 100 women randomized to directly observed HAART and 100 women randomized to self-administered HAART in each trial.

### Sensitivity Analyses

We evaluated our model assumptions and data inputs by performing univariate and bivariate sensitivity analyses. A plausible range was established for each parameter using the highest and lowest values in the published literature or confidence intervals when available, and by adopting alternative assumptions to those used in the base case analysis. When only one data point was available for a given parameter, we established plausible ranges through the use of expert opinion.

## Results

### Model Calibration

**Figure 2:**
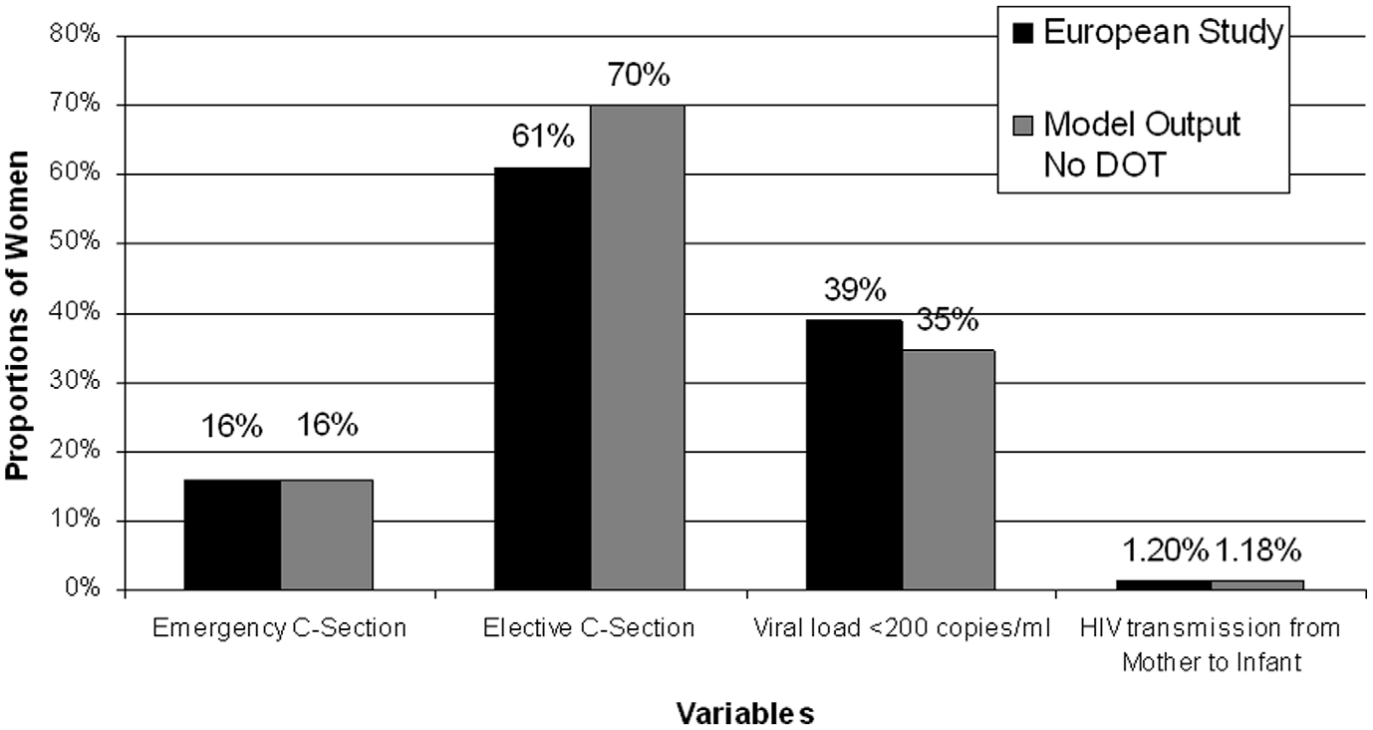
Model Calibrations. The model was calibrated to a 2005 European Collaborative Study that examined the effect of HAART on prevention of mother-to-child transmission in over 4000 mother-child pairs [33]. We calibrated the output of our model's self-administered therapy branch to the European study's results for proportion of women receiving an emergency C-section; proportion of women receiving an elective Cesarean section; proportion of women with an undetectable viral load (less than 200 copies/ml); and percent of HIV transmission to the infant.

Our final model was well calibrated to outcomes observed in a large European cohort study of pregnant women with HIV infection; we chose to calibrate our model to this study because it was the largest available study conducted in a context where HAART was widely available [33]. Final model parameter sets applied to the self-administered HAART strategy reproduced risks of mother-to-child HIV transmission, reduction in viral load in mothers receiving standard HAART, and risk of both emergency and elective Caesarean section reported in this study (**Figure 2**). The “no HAART” strategy in the model reproduced rates of vertical transmission consistent with those seen in trials of zidovudine and Cesarean section for prevention of HIV transmission [53], [54].

### Effectiveness and Cost-Effectiveness of Directly Observed HAART

**Figure 3:**
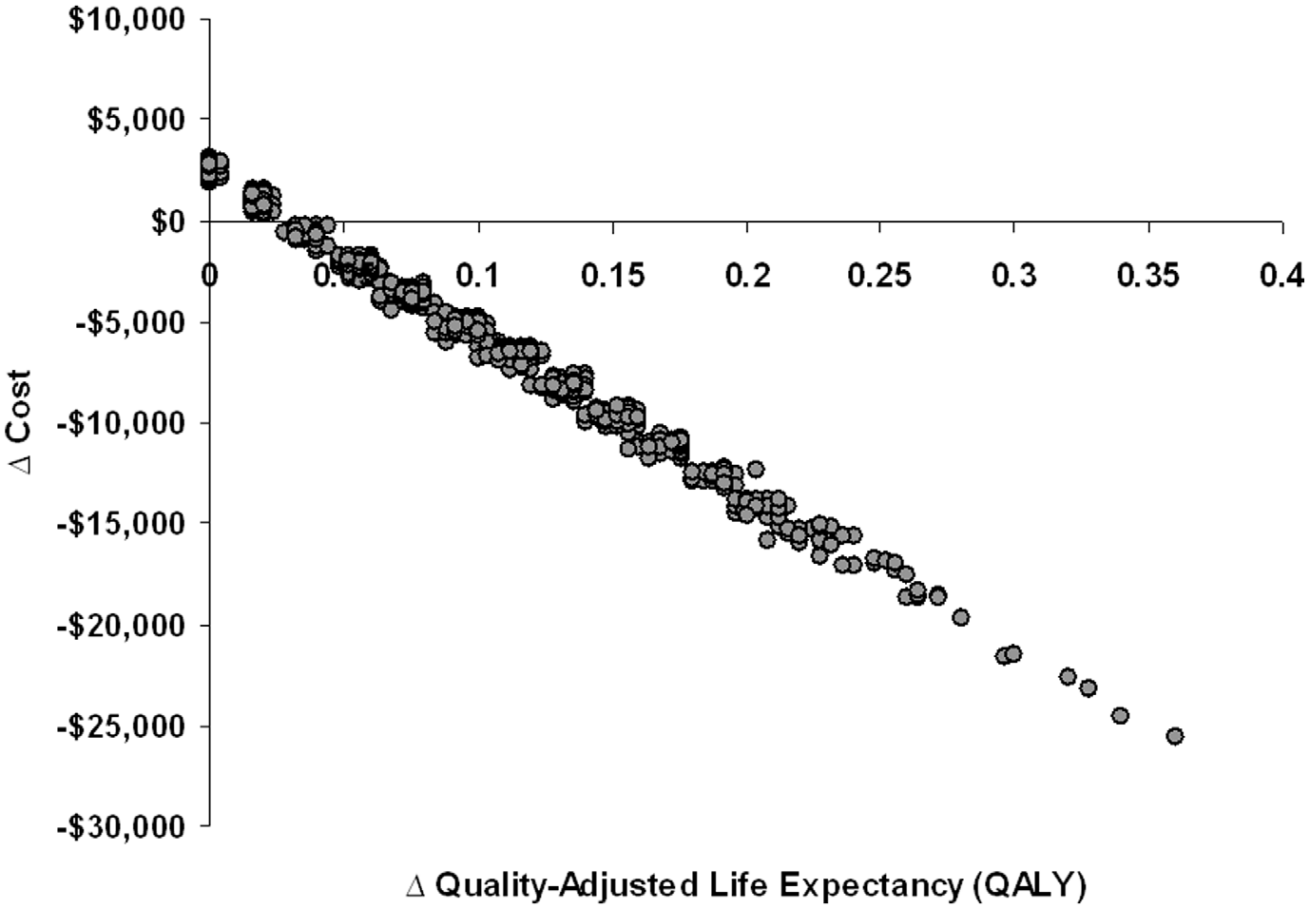
Projected Cost-Effectiveness of Directly Observed Highly-Active Antiretroviral Therapy in Pregnancy. The estimated cost-effectiveness of directly observed highly-active antiretroviral therapy (HAART) as compared to self-administered HAART. Each grey circle represents one of 5000 simulated trials comparing the two strategies, with 200 women in each trial. The incremental cost of directly observed therapy is on the Y-axis, while the incremental change in quality-adjusted life expectancy among infants is on the X-axis. Under base-case assumptions, directly observed therapy reduced costs and increased quality-adjusted life expectancy in most trials.

The outcomes of five thousand simulated randomized controlled trials of directly observed HAART, compared to self-administered HAART, are presented in **Figure 3**. While we did include a “no HAART” strategy in our model, this strategy resulted in increased net costs and reduced quality-adjusted life expectancy relative to the self-administered HAART and directly observed HAART strategies in the cohort as a whole, and thus would never be preferred.

A reduction in the probability of mother-to-child HIV transmission was seen with directly observed HAART in most trials, with a projected average relative risk of HIV infection of 0.39 among infants born to women receiving DOT versus those receiving self-administered HAART, and a relative risk of 0.09 as compared to women receiving no HAART (**Table 2**). The reduction in viral load among women receiving directly observed HAART also decreased the requirement for elective Caesarean section among women giving birth at term. There was a resultant average increase in the projected quality-adjusted survival of 11 quality adjusted life-days among infants born to women receiving directly observed antiretrovirals.

**Table 2:** Projected Average Cost and Effectiveness of Directly Observed HAART in 5000 Simulated Randomized Trials.

Strategy	Average Cost	% Requiring Caesarean Section	% Mother-to-Child HIV Transmission	Infant's Quality-Adjusted Life Expectancy	Incremental Cost-Effectiveness Ratio ($/QALY)
**All Trials**					
**Self-administered HAART**	$73,043	70.5	1.22	29.71	——
**HAART with Directly Observed Therapy**	$73,470	67.8	0.48	29.74	Highly cost-effective * ($14,233)
**No HAART**	$77,012	84.9	5.44	29.55	Dominated *

**NOTE:** HAART, highly-active antiretroviral therapy; QALY, quality-adjusted life years.

* A health care intervention is “dominated” if it costs more, but provides less health benefit, than a competing intervention. A dominated health intervention is never preferred [50]. A health care intervention is considered to be “cost-saving” when it costs less, but provides more health than, a competing intervention; “highly cost-effective” when it costs less than the GDP per capita; and “cost-effective” when it is between one and three times a country's GDP per capita. A cost-saving intervention is always preferred [49], [50].

On average, direct observation of HAART in the third trimester was projected to be a highly cost-effective intervention, with gains in quality-adjusted survival described above accompanied by net societal costs of $14,233. When trials were stratified according to participants' baseline viral loads, directly observed HAART was projected to be a cost-saving intervention in cohorts with viral loads above 1000 copies/ml. We projected savings of $54,278 per QALY in individuals with viral loads between 1,000 and 10,000 copies/ml, and savings of $26,240 per QALY in individuals with viral loads >10,000 copies/ml (**Table 3**).

**Table 3:** Projected Average Cost and Effectiveness of Directly Observed HAART in 5000 Simulated Randomized Trials.

Strategy	Average Cost	% Requiring Caesarean Section	% Mother-to-Child HIV Transmission	Infant's Quality-Adjusted Life Expectancy	Incremental Cost-Effectiveness Ratio ($/QALY)
**Average Baseline Viral Load <1000 copies/ml**					
**Self-administered HAART**	$70,462	67.8	0.44	29.75	——
**HAART with Directly Observed Therapy**	$72,213	66.5	0.13	29.76	$175,100
**Average Baseline Viral Load 1000–10,000 copies/ml**					
**Self-administered HAART**	$80,852	79.6	3.65	29.65	——
**HAART with Directly Observed Therapy**	$75,967	70.7	1.25	29.74	Cost-saving*
**Average Baseline Viral Load Greater Than 10,000 copies/ml**					
**Self-administered HAART**	$86,636	83.8	5.62	29.54	——
**HAART with Directly Observed Therapy**	$85,324	81.1	4.32	29.59	Cost-saving*

**NOTE:** HAART, highly-active antiretroviral therapy; QALY, quality-adjusted life years.

* A health care intervention is considered to be “cost-saving” when it costs less, but provides more health than, a competing intervention; “highly cost-effective” when it costs less than the GDP per capita; and “cost-effective” when it is between one and three times a country's GDP per capita. A cost-saving intervention is always preferred [49], [50].

### Sensitivity Analyses

We performed wide-ranging sensitivity analyses by varying parameter values and using alternate model assumptions (**Table 4**). The projected attractiveness of directly observed therapy was insensitive to plausible changes in most model variables, including the probabilities of vertical HIV transmission, prematurity, and emergency and elective Caesarean section; obstetrical costs; and future costs associated with HIV infection and prematurity. Projections were somewhat sensitive to the baseline effectiveness of HAART in pregnant women (i.e. drug effectiveness without directly observed therapy), and the effectiveness of directly observed therapy. Projections were highly sensitive to the enhancement of toxicity of HAART as a result of greater drug exposure with directly observed therapy.

**Table 4:** Selected Univariate Sensitivity Analyses of Directly Observed HAART Relative to Self-Administered HAART.

Variable	Discounted Quality Adjusted Life Expectancy (QALY), Self-Administered HAART	Discounted Quality Adjusted Life Expectancy (QALY), Directly Observed HAART	Cost-Effectiveness of Directly Observed HAART ($/QALY)
Baseline values	29.72	29.75	Highly cost-effective ($15,430)
Efficacy of self-administered HAART			
All women experience 2.0 log_10_ reduction	29.76	29.76	Dominated ‡
No sustained reduction with self-administered HAART *	29.69	29.74	Cost-saving
Reduction in viral load with directly observed HAART			
0.5 Log_10_	29.76	29.77	Cost-effective ($136,600)
2.4 Log_10_	29.72	29.76	Cost-saving
Probability of elective Caesarean section			
0.5	29.71	29.74	Highly cost-effective ($13,640)
0.7	29.72	29.75	Highly cost-effective ($15,270)
Probability of non-elective Caesarean section			
0.09	29.71	29.74	Highly cost-effective ($21,433)
0.21	29.70	29.73	Highly cost-effective ($7,033)
Probability of prematurity			
0.06	30.58	30.61	Highly cost-effective ($18,000)
0.27	29.29	29.32	Highly cost-effective ($15,100)
Probability of vertical transmission of HIV †			
Lowest	29.70	29.73	Highly cost-effective ($3,833)
Highest	29.74	29.76	Cost-effective ($66,850)
RR of drug toxicity with HAART			
Severe toxicity (Baseline probability 0.5%)			
2	29.70	29.71	Cost-effective ($46,400)
5	29.71	29.68	Dominated ‡
Moderate toxicity (Baseline probability 5%)			
2	29.58	29.46	Dominated ‡
5	29.57	29.00	Dominated ‡
Discounted quality-adjusted life expectancy § (HIV Infection/No HIV Infection)			
25/31	29.68	29.72	Highly cost-effective ($11,850)
30/31	29.76	29.77	Cost-effective ($59,100)
25/32	30.64	30.69	Highly cost-effective ($11,060)
30/32	30.71	30.73	Highly cost-effective ($18,900)
Antiretroviral costs, including DOT ¶			
Lowest	29.72	29.74	Highly cost-effective ($25,000)
Highest	29.71	29.74	Not cost-effective ($630,360)
Obstetrical costs ∥			
Lowest	29.72	29.75	Highly cost-effective ($20,833)
Highest	29.72	29.75	Highly cost-effective ($11,666)
Lifetime healthcare costs, pediatric HIV infection §			
$140,600	29.72	29.75	Cost-effective ($77,200)
$660,930	29.72	29.75	Cost-saving
Lifetime costs, prematurity §			
$214,200	29.73	29.76	Highly cost-effective ($11,300)
$407,400	29.71	29.74	Highly cost-effective ($16,333)
Discount rate			
0	74.64	74.79	Highly cost-effective ($3,360)
7	14.40	14.40	Dominated ‡
10	10.57	10.57	Dominated ‡

**NOTE:** HAART, highly-active antiretroviral therapy; QALY, quality-adjusted life years. Each estimate based on 10 simulated randomized trials with 1000 women per trial.

* Simulated through 0.75 log_10_ reduction in viral load in 65% of women, with 0.25 log_10_ response in the remainder.

† Highest probability of vertical transmission incorporated upper-bound transmission probability for each maternal viral load, and lower-bound estimate for effectiveness of Caesarean section, while lowest probability incorporated lower-bound transmission probabilities and upper-bound estimate for effectiveness of Caesarean section.

‡ A health care intervention is “dominated” if it costs more, but provides less health benefit, than a competing intervention. A dominated health intervention is never preferred [50]. A health care intervention is considered to be “cost-saving” when it costs less a competing intervention; “highly cost-effective” when it costs less than the GDP per capita; and “cost-effective” when it is between one and three times a country's GDP per capita, given that the intervention provides more health benefit than a competing intervention [49], [50].

§ Discounted to present value at 3% per annum.

¶ Incorporated upper- and lower-bound estimates for costs of highly-active antiretroviral therapy (HAART), peripartum zidovudine therapy, and delivery of directly observed HAART.

∥ Incorporated upper- and lower-bound estimates for costs of vaginal delivery and Caesarean section.

**Figure 4:**
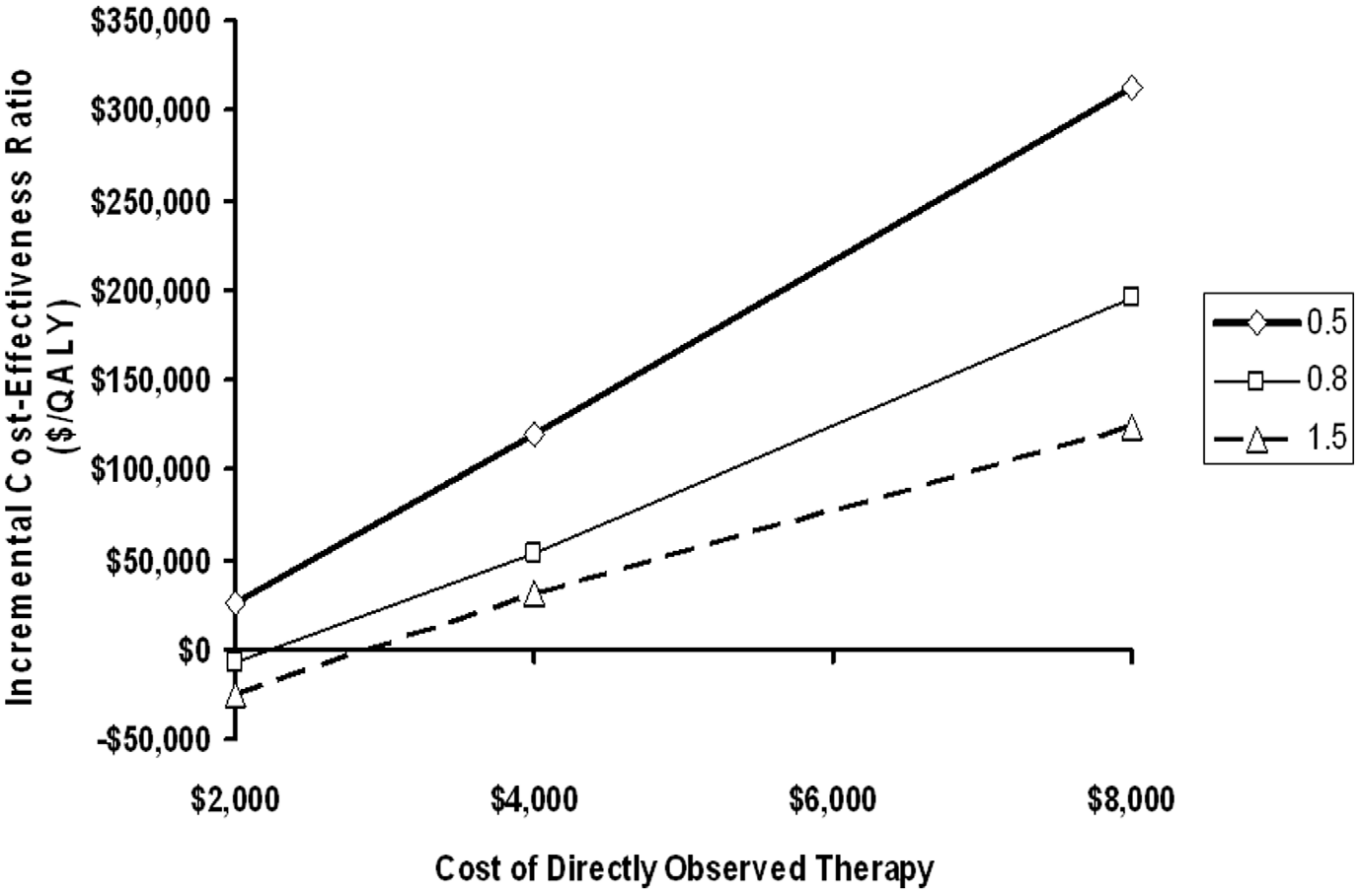
Two-Way Sensitivity Analysis on Effectiveness and Cost of Directly Observed Therapy. Each diagonal curve represents a different estimate of effectiveness (log_10_ reduction in viral load) of directly observed therapy: −0.5 log_10_ (thick line, squared); −0.8 log_10_ (thin solid line, diamonds); or −1.5 log_10_ (dashed line, triangles). Incremental cost-effectiveness of directly observed therapy relative to self-administered antiretrovirals appears on the Y-axis. At each level of effectiveness, increasing the cost of directly observed therapy increases the cost-effectiveness ratio associated with this intervention; however, only under the most unfavorable cost and effectiveness assumptions does this ratio exceed $50,000 per quality-adjusted life-year (QALY) gained, a commonly cited cost-effectiveness threshold (horizontal dashed line). Values falling below the X-axis indicate that directly observed therapy is cost-saving.

In our base case analysis, we assumed that the costs of direct observation of HAART would be due to personnel time required to deliver and observe administration of medications. However, creation of a novel program could be associated with other fixed and capital costs (office space and supplies, acquisition of vehicles, etc.). The results of two-way sensitivity analyses on the effectiveness and cost of directly observed HAART in pregnancy are presented in **Figure 4**. When we biased our analysis against directly observed therapy by increasing the cost of drug delivery four-fold, and by reducing the effectiveness of direct observation to a 0.5 log change in viral load, the cost-effectiveness ratio associated with this strategy was $313,000 per QALY, a ratio that exceeds a commonly cited threshold for cost-effectiveness [49]. However, this is an extremely unfavourable assumption [50], [55]. Most other combinations of DOT effectiveness and cost were cost-effective or cost-saving, relative to self-administered HAART.

**Figure 5:**
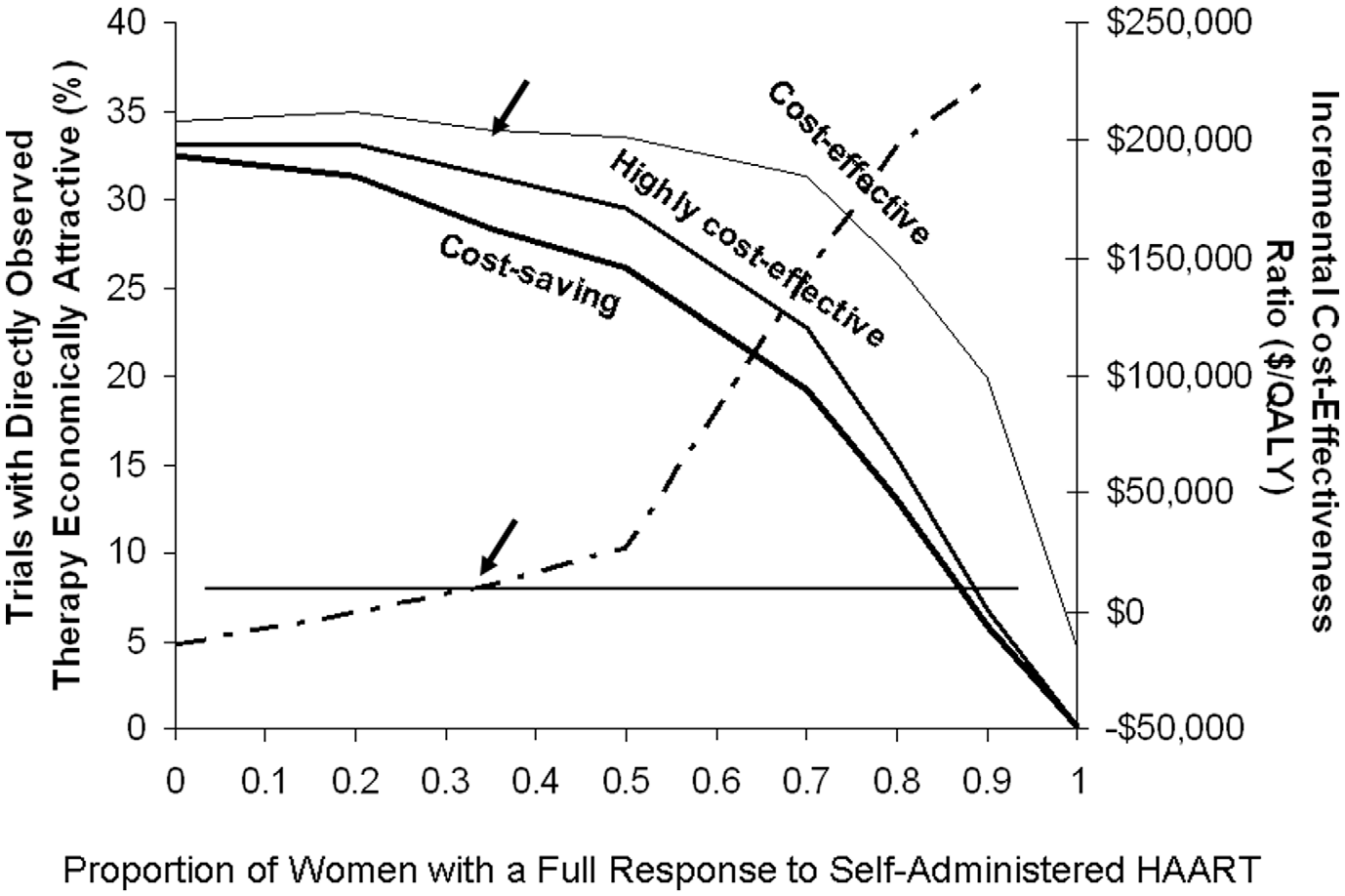
Sensitivity Analysis of Increasing Baseline Effectiveness of Self-Administered Antiretroviral Therapy. The proportion of women with a full response to self-administered antiretroviral therapy (i.e., 2.0 log_10_ reduction in viral load) is presented on the X-axis. The left-sided Y-axis indicates the proportion of 200-person clinical trials that find directly observed therapy to be cost-effective for various willingness-to-pay thresholds (thick black curve, WTP = $0; medium black curve, WTP = $50,000; thin black curve, WTP = $150,000). Average incremental cost-effectiveness ratios for directly observed therapy, relative to self-administered antiretroviral therapy (dark dashed curve) are presented on the right-sided Y-axis; values below $0 indicate that directly observed therapy is a cost-saving health intervention. As the proportion of women who have a full response to self-administered HAART increases, there is a decrease in the proportion of women for whom DOT is cost-effective. An increase in the willingness-to-pay threshold leads to an increase in the proportion of women who find this intervention cost effective. Arrows indicate base-case values.

We postulated that concern for a developing infant might result in enhanced adherence to self-administered HAART by pregnant women with HIV infection. We simulated such an effect by increasing the likelihood that pregnant women taking HAART would have a full (i.e., 2 log_10_) decrease in viral load with self-administered HAART (**Figure 5**). As baseline antiretroviral effectiveness of self-administered HAART improved, directly observed administration became less economically attractive at all tested willingness-to-pay (WTP) thresholds. The incremental cost-effectiveness ratio of DOT was cost-effective (under $50,000, a commonly cited cost-effectiveness threshold) as long as the proportion of women responding fully to self-administered HAART was under 0.6.

## Discussion

Mother-to-child HIV transmission remains an important but potentially preventable consequence of HIV infection in reproductive-age women. Although improvements in obstetrical management of HIV-infected women have greatly reduced the incidence of perinatal HIV infection, women now comprise 25% of those with newly diagnosed HIV infection and over 146,000 female adults and adolescents currently live with HIV/AIDS in the United States, suggesting that the pool of infants at risk for perinatal HIV infection remains large [1].

Based on the direct relationship between maternal viral load and risk of vertical transmission, and the potential impact of directly observed therapy on viral load, we hypothesized that the provision of directly observed antiretroviral therapy to pregnant women with HIV infection could result in substantial health and economic gains, including a diminished need for Caesarean sections, decreased mother-to-child transmission of HIV, and increased quality-adjusted life expectancy in infants born to HIV-infected women. By diverting the large downstream medical costs associated with pediatric HIV infection, the direct observation of HAART in the third trimester of pregnancy was projected to be a highly cost-effective health intervention from a societal perspective, notwithstanding the short-term costs associated with drug delivery and directly observed drug administration, and targeting DOT to women with viral loads >1000 copies/ml on self-administered HAART was projected to be cost-saving. It is notable that cost-saving health interventions (such as vaccination against diseases of childhood) are uncommon in healthcare; most currently utilized healthcare interventions increase total costs, but are considered cost-effective if they provide a corresponding increase in health [50], [55]. As the Commission on Macroeconomics and Health of the World Health Organization has suggested that interventions can be considered cost-effective if they provide life years at a cost of <3 times per capita gross national income (formerly gross domestic product), our analysis suggests that DOT-CART would be cost-effective not only in high-income countries, but in a large number of middle-income countries as well [49], [56].

While our analysis would strongly support the use of directly observed HAART in women in the third trimester of pregnancy, we may have underestimated the potential health benefits of this intervention. Direct observation of HAART could enhance the health of infants who acquire HIV infection by reducing the probability of primary infection with resistant virus [57], and could also improve future health in the mother by preserving the potency of antiretroviral classes for future use [6]. DOT might be given to those women with particularly unsettled social situations who would be most unlikely to benefit from self-administered HAART, such as those who are homeless, have substance abuse issues, or mental illness. Furthermore, the establishment of structured contact between pregnant, HIV-infected women and public health personnel, as would occur in the context of directly observed antiretroviral therapy, has been associated with improved pregnancy outcomes [58]. Such contacts could link infected women to improved healthcare after delivery as well as introduce them to more healthful behaviours while pregnant and perhaps more open to behaviour change.

It is notable that the favourable health and economic projections associated with directly observed HAART in pregnancy remained robust even in the face of extreme variation in cost and effectiveness of directly observed therapy. We were able to identify only one scenario in which direct observation of HAART in pregnancy would not be preferred to self-administered HAART: a situation in which over 60% of pregnant women responded maximally to self-administered HAART, one that is inconsistent with empiric data on the high-risk women who are the focus of this analysis [14].

Like any mathematical model, the model we present here is a simplified representation of complicated systems, and is thus subject to limitations. In particular, a relatively simple model is limited in its ability to capture the all the complexities related to the clinical management of HIV in pregnancy; thus, models such as this one are not a substitute for randomized clinical trials. However, by synthesizing the best available data on antiretroviral use in pregnancy and the potential benefits of directly observed antiretroviral therapy, modeling can help to inform clinical practice while such trials are pending. Also, by demonstrating the large potential benefits of a novel approach to therapy, such modeling may help to mobilize interest in, and support for, such trials. Our model is particularly important given that this topic is so difficult to study prospectively, due to the small number of HIV-infected infants born in the U.S. Multi-centre clinical trials are an important tool for better defining maximally effective and cost-effective interventions for prevention of vertical HIV transmission, especially with mothers at risk of medication non-adherence.

In summary, based on best available data, we projected that pregnancy provides a unique indication for the use of directly observed antiretroviral therapy for HIV infection. In this context, a modest investment of resources would result in substantial health benefit with a net reduction in societal costs as a result of decreased mother-to-child HIV transmission. Randomized controlled trials of directly observed therapy can be advocated as a reasonable next step in optimizing the prepartum care of HIV-infected women.
